# A New Paradigm Is Needed for Medical Education in the Mid-Twenty-First Century and Beyond: Are We Ready?

**DOI:** 10.5041/RMMJ.10152

**Published:** 2014-07-25

**Authors:** Dan E. Benor

**Affiliations:** Professor Emeritus, Faculty of Health Sciences, Ben-Gurion University, Beer-Sheva, Israel

**Keywords:** Medical education, new paradigm, twenty-first century

## Abstract

The twentieth century witnessed profound changes in medical education. All these changes, however, took place within the existing framework, suggested by Flexner a century ago. The present paper suggests that we are approaching a singularity point, where we shall have to change the paradigm and be prepared for an entirely new genre of medical education. This suggestion is based upon analysis of existing and envisaged trends: first, in technology, such as availability of information and sophisticated simulations; second, in medical practice, such as far-reaching interventions in life and death that create an array of new moral dilemmas, as well as a change in patient mix in hospitals and a growing need of team work; third, in the societal attitude toward higher education. The structure of the future medical school is delineated in a rough sketch, and so are the roles of the future medical teacher. It is concluded that we are presently not prepared for the approaching changes, neither from practical nor from attitudinal points of view, and that it is now high time for both awareness of and preparation for these changes.

## INTRODUCTION

The twentieth century was a very prolific period for medical education. The slow progress that characterized the previous eras, from the Renaissance until the end of the nineteenth century, changed pace, and educational innovations were introduced at an ever growing rate. To mention just a few conspicuous milestones among many, one must acknowledge the Flexner report^1^ that placed medical education upon sound scientific foundations; Case Western Reserve University at Cleveland, Ohio, USA, that originated the integrated organ systems approach which enables meaningful interdisciplinary learning of themes rather than scattered disciplinary factual knowledge;^2^ McMaster University at Hamilton, Ontario, Canada, that introduced the problem-based learning which enhances self-directed learning of multi-faceted patient problems by individuals and groups of students in an integrative way;^3^ Ben-Gurion University at Beer-Sheva, Israel, that developed an intensive early clinical exposure from the onset of medical studies^4^ and a far-reaching faculty development system;^5^ the creation of the Objective Structured Clinical Examination (OSCE) by Harden et al. at Dundee, Scotland,^6^ that enabled for the first time a reliable and valid measurement of students’ clinical skills; and, finally, “the Second Track,”^7^ initiated at the University of New Mexico at Albuquerque, USA, that enhanced considerably the acceptance, even by the conservative educational establishment, of the innovative methods. All these innovations, and many more, were adopted, fully or partially, by a growing number of medical schools in all the five continents, including schools in developing countries.^8^,^9^ Actually, towards the turn of the century and the first decade of the twenty-first century, reforms of the undergraduate curriculum have become the conventional rather than the pioneering phenomena.

Moreover, the twentieth century witnessed the emergence of medical education as a recognized medical discipline, if not a profession. Medicine owes this transformation to a handful of pioneers who dared to challenge the conventional medical education. The initiatives of these individuals were based upon new thinking about education in general that was voiced during the second half of the century^10^–^14^ and on better understanding of the neuropsychological basis of the learning process.^15^,^16^ These, together with growing dissatisfaction with contemporary medical education,^17^ brought about the formulation of a set of recommendations made by national and international organizations such as the American Association of Medical Colleges (AAMC),^18^,^19^ the World Health Organization (WHO),^20^ and the World Federation for Medical Education.^21^ All of them proposed somewhat similar lines along which medical education should develop. These guidelines included, among other recommendations, the following: to set institutional objectives; to centralize the control of resources, including curricular time and content; to refocus the orientation from teachers to students; to replace the traditional biomedical model, focusing on cure of diseases, by the bio-psychosocial model^22^ that focuses also on environmental, social, psychological, and familial factors which have bearing upon health; and to promote preventive medicine and health education of the population at large. Also recommended were profound changes in the teaching–learning methods, moving from passive to active learning; aiming at understanding principles rather than memorizing facts; reshaping the curriculum around interdisciplinary themes; exposing students to clinical reality as early as possible and emphasizing problem-solving, clinical reasoning, and critical thinking capabilities.

Although these recommendations were fully adopted only by a few dozen medical schools, many more institutions adopted some of them and adapted others to their own institutional culture. These new approaches, therefore, have had a profound influence on medical education worldwide. The dissemination of the new educational methods was further accelerated by the financial support from the Robert Wood Johnson Foundation^23^ and the recently launched National UME-21 Project.^24^

Yet, all these changes occurred within the basic framework that Abraham Flexner prescribed,^1^ namely: pre-medical studies of natural and social sciences followed by two years devoted entirely to basic medical sciences and another two years spent in rotating clerkships in teaching hospital departments, supplemented by a yearlong internship. In the USA the pre-medical education takes place in a college, followed by four-year medical school, while in most of the other parts of the world the pre-med studies comprise the first phase of a five- or six-year medical school.

In order to prepare the graduates for the twenty-first-century medical practice, many medical educators and educational researchers continue to call, even today, for further implementation of curricular and structural reforms, believing in evolutionary development of medical education.^25^,^26^ However, the traditional framework is challenged nowadays by technological, medical, and social developments that change the world in which we are living. The changes occur at an almost exponential rate, approaching a singularity point.^27^ Further evolution of medical education may not be possible anymore, and an entirely new paradigm will be required. The evolution, so it is assumed, will be replaced by revolution.^28^

The present paper will try to identify the main factors that may force a revolution upon medical education. It will further try to analyze the impact of each, to draw a rough draft of the future medical school, to delineate the roles of the future medical teacher, and to underline the difficulties that the present generation of medical teachers may encounter during the transition phase.

## THE INFORMATION REVOLUTION

Tomorrow’s students will need neither classrooms nor flesh-and-blood lecturers, nor books and libraries, in order to get scientific or clinical information.^29^ Actually, world-class information presented by the best presenters from the world’s leading institutions is already at their fingertips anywhere, anytime. The availability and accessibility of information has been growing at an exponential rate during the last two decades through the world wide web, home, mobile, palm, and tablet computers, smartphones, and soon enough by wearable devices such as glasses, wrist “watches”, and others, and perhaps by implanted, under-skin chips. It will be unwise, if not stupid, to continue to transfer information in auditoria to passive audiences who do not need it. It is also cost-ineffective, in an understatement.

The developing technology will not only be able to provide the learners with appropriate resources, but will also be able to follow the progress of each one of them individually, identifying lacunae in the knowledge and offering remedial teaching. Moreover, individualized self-examinations will become powerful learning tools.

However, the vast and growing amount of information requires the learners, and will require them more and more, to be selective, to assess the quality of the information, and to pick the reliable and sound sources upon which they can build their knowledge base. This is almost like finding a needle in a haystack. Furthermore, in order to make the learning effective, the students will have to set priorities so that new information will be interwoven into existing networks acquired by previous learning, as the cognitive sciences postulated years ago.^15^ The main role of the future medical teacher will, therefore, be to teach the students to retrieve, select, assess, and prioritize information. Are we ready for this?

But acquiring factual knowledge is only the basic step of the learning process. More important, though complicated, is to gain problem-solving, critical reasoning, and clinical judgment capabilities. Information technology may and will assist in acquiring these cognitive skills, yet the main burden of teaching will remain an obligation of the future medical teachers, at least in the nearer future. Teaching these skills is within the scope of medical teachers’ responsibility for a long period of time. However, this teaching is taking place in conjunction with the factual content that they are teaching in their courses. Will they be able to address these higher cognitive skills while they are neither the source of the information nor the authority in regard to it?

## SIMULATIONS

So far the discussion has related to the cognitive domain. However, technology is changing education also in the psychomotor domain by offering more and more sophisticated simulators as well as creating virtual reality.^30^ Students will not have to acquire clinical manual skills for the first time during clerkships, and residents will not have to gain experience only through years of practice, sometimes risking patients’ safety. Instead, simulation labs will enable learning manual skills that pertain to a great variety of conditions, in a safe environment and at the learner’s pace. They will also identify mistakes in performance, correct flows, and enable repetitions until mastery of the skill is achieved. Operating robots, activated by remotely situated surgeons, already exist.^31^ These and better technologies will become commonplace in education as well.

An inevitable byproduct of this development will be a significant shortening of clerkships and residencies and great expansion of self-learning in simulation labs. Medical teachers will have to work together with computer programmers and technical experts to create the needed variety of virtual conditions rather than to teach students. Are we ready for this?

## MEDICAL PRACTICES

Hospitals will never disappear. However, the patient mix in the hospital is already changing due to the rising life expectancy and aging of the population.^32^ Also, there will be a growing prevalence of low-severity diseases that will be, and actually already are, treatable at community-based medical facilities and at patients’ homes. The hospitalized patients will thus be older, suffer from a smaller variety of diseases, in more complex conditions.^33^ This reduces the repertoire of conditions to which students are exposed during their study period in hospitals. Moreover, the duration of the hospitalization is getting shorter and shorter. This is because of financial considerations, medical and pharmaceutical developments, and improvement of the community-based health care, supported to a large extent by technology. Students will not only experience fewer medical conditions in hospitals, but also will not be able to perceive the natural history of the disease and may be ignorant as to its later stages after the short hospitalization. Individualized medicine, based upon genetic profile of the patients, will decrease further the hospitalization rate and duration, and will diminish further the clinical inventory of the students. Larger parts of the clinical teaching, therefore, will inevitably move outside the teaching hospital wards into outpatient clinics, community-based clinics, nursing homes, hostels, patients’ homes, and other facilities that may emerge in the future.^34^ Are the health care-providing organizations ready for this new educational responsibility that they are about to confront? Would the medical schools adopt community-based staff as equally appreciated, treated, and rewarded teachers?

The changes occurring in medical practice include also a growing need of team work.^35^,^36^ The mid-twenty-first-century physician will have to know more, to master more technical skills in order to operate more sophisticated technology, and to treat older and more complex patients. She or he would not be able to do it alone. Teaming up with health professionals other than doctors as well as with technicians and other staff is inevitable. Yet, the contemporary medical students are not exposed to these other medically related professionals and often do not trust them.^37^,^38^ Future medical education will be required to provide interprofessional education in which small interdisciplinary groups will try to solve patients’ problems, mainly during the clinical studies but also beforehand. Moreover, the medical profession will have not only to accept partnership with other health-related professionals, but also to agree to rotating leadership roles according to the problem at hand. The physician in the team should not always be the leader. Would the medical profession be able to embrace other professionals in such a way? Would universities accept them as teachers?

## SOCIOLOGICAL AND SOCIAL FACTORS

The societal regulatory processes lag behind the technology and are ambivalent with regard to a variety of issues raised by the developments. Some of these are everyday issues, well known to everybody, ranging from self-inflicted loss of privacy to the diminishing face-to-face communication between people, especially among the younger generation. There is also no consensus with regard to new forms of family, racial and feminist issues, and many others.

Mid-twenty-first-century medicine will pose, and actually already poses, a long list of additional issues for society on which consensual agreement has not been yet achieved and that medicine and bioethics did not yet decide upon. These include artificial insemination and host mothering, cloning, gene manipulations, stem cell research and its use for therapy, and in the near future perhaps also selection of the gender of offspring, artificial womb, delay or prevention of aging, as well as assisted suicide and euthanasia, to mention just a few. It well may be that prostheses operated by will of amputees,^39^ devices that enable blind people to see,^40^ and technological intervention in depression,^41^ which all are blessed developments, may be the first attempts of intrusive intervention in the brain functions. This may lead to far-reaching and untoward territories and will pose a new array of ethical dilemmas to the society. The future will undoubtedly bring about many more, including human–machine hybrids and decision-making and even feeling machines.^27^

The impact of all these upon medical education will be dramatic. Both students and faculty will be asking difficult questions, which no on-line information, available as it may be, can fully answer. Moreover, these moral and ethical dilemmas may cause much distress to all involved—students, teachers, patients, and families.

The future medical teacher will have to address these ethical dilemmas. Contrary to the cognitive domain, in which the medical teacher will lose much of his or her authority, in this minefield of the affective domain, he or she, willingly or unwillingly, will become the only accessible authority for the student. This means more than just being able to address the various issues. It will also require basic skills in dealing with rough emotional conditions, both of students and patients. Are we ready for this new task? Will we be willing to undertake this drudgery?

The societal and social factors impose yet additional burdens upon medical education. At the midst of the previous century a considerable proportion of the applicants to medical schools was driven by altruism, wishing to help people in need. A smaller, although still significant, proportion of them maintain this throughout their career. In the last two decades the driving force to study and practice medicine has become self-fulfillment.^42^ The individual is both the center and the aim, and the medical school and its teachers are expected to enable development of the full potential of the individual. This, together with the information explosion, deposed the physician and the medical teacher from the pedestal upon which they were standing for centuries, to the street level. This process was augmented by introducing student, rather than teacher, orientation that was recommended so emphatically by all the international forums mentioned above.^17^–^21^,^23^ It is envisaged that this process of entitlement will continue at an ever growing pace during the twenty-first century. Medical teachers will be perceived as providers of a service and therefore obliged to the student-client by the same process that views the physician as a provider of service to a client, formerly known as a patient.^43^ The language used today by students in the discourse with their teachers, as compared to the language used several decades ago, may hint to what is to be expected in the future. Are we willing to accept this?

The societal factors may have still another aspect which is of a crucial importance to medical education. This is an intervention in the school’s affairs by societal agencies, such as governments, driven by public opinion, aiming at controlling priorities, procedures, and even contents. If this presently seems unimaginable to many, one can look upon what happened to the professional freedom of physicians in the era of managed care.^44^ It can be predicted that the unconditional victory of neoliberalism in large parts of the world will inflict the same fate on higher education, including medical education. Universities and health care-providing organizations already feel restriction of their budgets. The pressure to cut costs of medical education and to limit its expenditure will undoubtedly increase as the time goes by.

Another cause for governmental and societal intervention in medical education will probably be the geographic maldistribution of health force, leaving large underserved populations. Still another is the improper distribution of physicians among the various clinical professions, expressed in an ever growing rate of specialization and a diminished proportion of generalists and especially of family practitioners. Some efforts made recently have slowed down this course of events,^23^,^24^ but it is very doubtful if the process will be reversed.

The control of society over medical education will, most probably, be through budgeting. Schools will compete for budgets and for enrollment of students, and will adapt their practices, including the curriculum, to the public demand. The sacred academic freedom will probably be maintained in research, but not necessarily in education. Are we ready for “managed education”? Are we aware of the path we are traveling?

## THE FUTURE MEDICAL SCHOOL

The medical school of the mid-twenty-first century will have fewer classrooms, if any, and auditoria will be used only for ceremonies and conferences. It will contain many more self-learning facilities, including digital libraries accessible from home, large simulation laboratories, teleconferencing installations, and many small seminar rooms for study groups. It will not become “a school without walls,” which means without a building, as some predict,^45^ since the research laboratories of basic and clinical scientists will remain and possibly expand, and the administrative functions will still be needed. However, the physical and conceptual proximity to teaching hospitals will become redundant.

Students will spend most of their time in self-learning, either individually or in small groups (some of them interdisciplinary), or both, and will follow individually designated paths based upon their progress as judged by frequent self-examinations prescribed by computers. The self-learning will be based on clearly spelled out detailed curricular objectives for each phase and every theme or subject matter.

However, studies that are based solely or mainly upon self-learning will require rigorous periodical summative evaluations, since the school will have diminished control over what the students have learned. Future medical education will, therefore, develop valid and reliable examinations, both written (actually, computerized) and simulation-based, that will allow the student to move from phase to phase, including, of course, the final and certification examinations. Such a comprehensive and rigorous evaluation system does not presently exist, although the psychometric science has made huge progress in the last few decades. Very few individual schools will have the resources and the expertise to develop such a large number of high-quality examinations. It is envisaged, therefore, that composing examinations will become a subspecialty of medical education, and that each such group of specialists, working together with medical experts, will serve a large number of schools, both nationally and internationally.

A course of studies that is based on self-learning, combined with unyielding rigorous evaluation, may make redundant the traditional division into semesters and years. The future medical student will be able to pace his or her own studies, to take periodic summative examinations whenever she or he feels ready, and to move to the next phase regardless of his or her classmates. Actually, the terms “class” and “classmates” may become history. There will be no four-, five-, or six-year medical schools. What can take four years for one particular student may take seven for another. Since the studies are based on self-learning and simulations, such self-pacing should not increase the school’s expenses to any noticeable extent. It should be stressed, however, that this does not relate to the clinical studies, in which the exposure to each medical discipline will remain mandatory and controlled, although shorter than today, and carried out in a variety of sites, as was mentioned above. Moreover, preventive medicine will become one of the major thrusts of the future medical curriculum. Each student will keep a logbook that will ensure that she or he has encountered all the listed conditions and performed all the specified activities, from interviews and physical examinations to diagnoses and interventions, to patient education and acknowledgment of and involvement in environmental health and prevention of diseases. Conditions that the student did not confront in the hospital he or she will have to meet or perform in one of the other sites before graduation in order to be allowed to take certification exams.

Such a medical school dramatically deviates from what we know today. It will require a major shift of the mental construct we presently have on what is a medical school. Are we ready for such a shift?

## THE FUTURE MEDICAL TEACHER

Medical teachers in the mid-twenty-first century will maintain some of their traditional roles. These include first and foremost being a role model for the student. This is especially true for clinical teachers. Along the entire history of mankind, role modeling is the single most powerful component of education at all levels.

The future medical teacher will also continue to “translate” the theoretical knowledge that the students acquired during their studies into clinical reality, and to interweave basic sciences information into clinical context. The students will be expected, as they are today, to apply their knowledge, at first in an “armchair” atmosphere, in which they are allowed to make mistakes and correct them in a protected environment and without the everyday pressure of time, which gradually grows as the student assumes more and more responsibilities. Finally, the future teachers will have to continue to teach, or, actually, assist the students to learn problem-solving, critical reasoning, decision-making, and clinical judgment as they are doing today, or perhaps even more. They will also start it at an earlier phase in the course of studies. Furthermore, teaching these skills will have to be more conscious and targeted and less intuitive and based upon tacit knowledge^46^ as it is today.

Preparing medical teachers for the traditional tasks will continue to be by the same training methods used today, such as short workshops and seminars, longer formal courses on pedagogy and didactics, mentoring, teaming up with a peer and others,^47^,^48^ as well as new training methods that should, and probably would, develop. However, the teacher-training and faculty development programs, which today are mostly voluntary and scattered, will become both obligatory and systematic. In the “managed education” era schools will not allow themselves to ignore student satisfaction and societal approval, as was discussed before.

However, the mid-twenty-first-century medical teacher will have also to teach the art and science of information management such as retrieval, assessment, and prioritization, as was described above. This role is not trivial. Medical teachers of today are “immigrants” to the digital world, while their students are “natives.” Indeed, most of the present teachers are able to “speak” the digital language to some extent, and can relatively easily find resources for their own research and publications. For their student, however, it is a “mother tongue.” As the older generation of teachers is fading out and the students of today will become teachers, this problem will vanish. During the transition period, however, this may become an issue that will require yet unknown methods of training. The task of teaching information management may be especially hard for the future teachers, since they will not be responsible for the content and the pace of learning. No doubt that it is easier to teach information management when it is conjunct with subject matter that the teacher teaches at the time.

The second new task of the future medical teacher will be to address ethical and moral dilemmas and to deal with emotional distress of students and patients in relation to these dilemmas, as was also discussed above. Presently, medical teachers have no skills of dealing with emotional conditions, and they do not perceive them to be in the realm of their responsibilities, leaving it to either psychologists, psychiatrists or ethicists and moral scientists. Moreover, the majority of medical faculty members nowadays look upon such experts as foreign to “real” medicine. Yet, emotional support will become soon enough one of the main trusts of the future teacher, and, therefore, a part and parcel of his or her training programs.

Contrary to these expansions in the role of the future medical teacher, there will be some reliefs, such as waiving the need both to transfer information and to evaluate the student’s cognitive and manual performance. Although the teacher will still have to provide the student with appropriate feedback on his or her clinical performance, the rest of the tedious task of evaluation will be transferred either to on-line personalized formative evaluation or to groups of evaluation experts for summative evaluation.

In the light of the above discussion, it is expected that a new genre of medical teacher will emerge, that will work side by side with the clinical teachers known today. This type of teacher will have to be a compassionate, tolerant, and indulgent companion to a group of students, kind of a mentor, a coach, and a guide. She or he will have to assist students to self-learn and direct them in information management, but also to consult, console, mirror behaviors, and suggest alternatives. This job description exceeds by far the present adviser’s role, and it will require much more time than the present medical teacher devotes to his or her students.

Each school will have to have such teachers, although not many. Who will be these teachers? First, they may, but do not have to be, specialists in any medical field. They may be of any age, gender, and seniority. They will be mainly, or even exclusively, clinicians in order to be able to address the issues that they are required to tackle. Most important—they must have free time for their educational job. Busy clinicians, therefore, will not be qualified for this role. Instead, these teachers will emerge from four sources: One is clinicians who wish to change their career path in the same manner that some clinicians, for example, move to managerial posts in the health care services or to public health functions. The second source of such teachers is clinicians who wish to take a break from their erosive clinical tasks, the same way that some are now taking a year or two off to indulge in research or embark upon some irrelevant studies to satisfy a specific interest or just to take a pause. The third origin of this kind of medical teachers will be retired physicians that were either forced to retire because of their age or chose to retire because of a variety of personal reasons. The last, and probably the smallest source of such medical teachers, will be young physicians who, during their residencies or after, will choose the educational path as a career.^49^

At a first glance this prediction may look highly improbable if not a sheer absurdity. At present, it is certainly out of consensus. But if it is judged in the context of mid-twenty-first-century medical education, in which schools will make great efforts to stay relevant for students and responsive to public demands, and in which the learning–teaching process will change radically, this idea ceases to sound strange. It requires only a shift in the psychological construct on education. Such a shift seems possible, even probable, once education becomes as important and lucrative as management, public health, and clinical research. Yet, are we ready for it?

## SUMMARY AND CONCLUSIONS

The present article attempts to describe the medical school of the mid-twenty-first century and beyond, its features, structure, and teachers. The predictions are based upon analysis of the current developments and on the visible trends in technology, sociology, and medicine. However, “It is difficult to make predictions, especially about the future,” said Nobel Prize laureate Niels Bohr (although some attribute this saying to others).^50^ The author admits that it is likely that not all the predictions presented in this paper will materialize exactly as they are described, and some may not occur at all; that the changes will not occur at a snap of a finger but rather be gradual over a considerable period of time; and that some of the described changes may affect, at the beginning, only part of the schools and only in some areas of the world before they will become the commonplace.

However, whether the changes will be profound or partial, occur sooner or later, be worldwide or limited to the developed countries—they are coming, and we are not ready for them, neither on the operational level nor on the conceptual one.

Several steps can and should be taken right now in order to be prepared for the mid-twenty-first-century medical education. First, we ought to train the teachers of today both how to master information management and how to teach it to their students. Second, we have to encourage students to seek information on their own, and learn to trust their self-learning. Then we have to expand learning by simulators, to invest in the developing equipment, and to educate tutors to use it for teaching.

However, the most significant and difficult component of preparedness for the changing medical education is attitudinal. Painful and excruciating as it may be, we have to realize that the Gordian knot tied between medical education and hospitals is loosening. This entails preparation of out-of-hospital sites for teaching, making the necessary arrangements with the health care-providing organizations regarding these sites, training the local interprofessional personnel, and— perhaps the trickiest—accepting them as equal partners who are equally rewarded.

Further, preparedness for the coming changes requires acknowledging educational activities as a scholar enterprise. Education cannot remain a side engagement of basic researchers and clinicians. Change in the status of medical teachers may well be the engine that will smooth the transition to the new paradigm.

One can suggest other measures that will be gradual and will not provoke destructive antagonism. Doing nothing, however, is not an option.
